# Multi‐omics integration in Caribbean Hispanics identifies cardio and cerebrovascular pathways in AD

**DOI:** 10.1002/alz.095342

**Published:** 2025-01-09

**Authors:** Jaclyn M. Eissman, Min N Qiao, Vrinda Kalia, Marielba Zerlin‐Esteves, Dolly Reyes‐Dumeyer, Angel Piriz, Saurabh Dubey, Renu Nandakumar, Annie J. Lee, Rafael A. Lantigua, Martin Medrano, Diones Rivera Mejia, Lawrence S. Honig, Clifton L. Dalgard, Gary W Miller, Richard Mayeux, Badri N. Vardarajan

**Affiliations:** ^1^ Taub Institute for Research on Alzheimer’s Disease and the Aging Brain, Columbia University, New York, NY USA; ^2^ G.H. Sergievsky Center, Columbia University, New York, NY USA; ^3^ Mailman School of Public Health, Columbia University, New York, NY USA; ^4^ Vagelos College of Physicians and Surgeons, Columbia University, New York, NY USA; ^5^ Pontificia Universidad Católica Madre y Maestra, Santiago, Santiago Dominican Republic; ^6^ CEDIMAT, Plaza de la Salud, Santo Domingo, Santo Domingo Dominican Republic; ^7^ Physiology & Genetics, Uniformed Services University of the Health Sciences, Bethesda, MD USA; ^8^ The American Genome Center, Center for Military Precision Health, Uniformed Services University of the Health Sciences, Bethesda, MD USA

## Abstract

**Background:**

Multi‐omics integration can clarify molecular mechanisms contributing to Alzheimer’s Disease (AD). We conducted a quantitative trait locus (QTL) analysis across three omics layers to identify genetic variants that regulate metabolomics, gene expression, and DNA methylation in AD.

**Method:**

We analyzed data from Caribbean Hispanic individuals from the Dominican Republic and New York with AD or family history of AD including: N = 750 with whole genome sequencing (WGS), RNA‐sequencing, and DNA methylation (in blood), and N = 272 with untargeted metabolomics. Metabolites (N = 5,883) were measured using liquid chromatography coupled to high‐resolution mass spectrometry. WGS data (7,588,678 variants) were normalized, aligned, and filtered for quality (BCFTools). Variants with a minor allele frequency greater than 5% were retained. The MatrixEQTL package in R was used for QTL analyses, adjusting for age, sex, and principal components for population substructure. Significant QTLs were declared at a false‐discovery rate of 0.05 across all tests. Pathway analyses (clusterProfiler) and colocalization with known AD SNPs were completed to identify causal variants and genes in AD.

**Result:**

19,336 SNP‐metabolite combinations were statistically significant after adjusting for multiple comparisons (FDR<0.05). 16,421 unique SNPs were identified as QTLs for 60.9% of all metabolites. 68% of FDR‐significant metabolomics QTLs overlapped with trans‐expression QTLs with P<10^−5^. The most significant associations were between the metabolomics QTLs (P.FDR<0.05) and cardiovascular pathways including brain ischemia, angiogenesis, and cerebrovascular disease; neuronal pathways including synaptic organization/signaling, neuronal apoptosis, neurogenesis, gliogenesis, and axonogenesis; and AD endophenotypic pathways including cognition, learning and memory, amyloid‐beta binding, and dementia. We additionally colocalized SNPs in the Bellenguez et al. 2022 AD GWAS (P<10^−3^) with metabolomics QTLs (P.FDR<0.05), identifying 24 unique colocalized genes. Notably, *SPOCK3* associated with verbal memory (Debette et al., 2015), *WWOX* associated with Aβ42/Aβ40 ratio (Stevenson‐Hoare et al., 2023), and *CACNA2D3* associated with an AD subgroup (Mukherjee et al., 2018).

**Conclusion:**

We identified common genetic variants that regulate metabolite levels, many of which were found to overlap with known AD variants and were enriched in AD‐relevant biological pathways. Our next steps include integrating QTLs of transcriptomic and epigenetic data to identify shared molecular pathways that underlie several omics layers leading to AD.

